# A pilot open series of lamotrigine in DBT-treated eating disorders characterized by significant affective dysregulation and poor impulse control

**DOI:** 10.1186/s40479-017-0072-6

**Published:** 2017-10-08

**Authors:** Mary Ellen Trunko, Terry A. Schwartz, Laura A. Berner, Anne Cusack, Tiffany Nakamura, Ursula F. Bailer, Joanna Y. Chen, Walter H. Kaye

**Affiliations:** 1 Department of Psychiatry, University of California, San Diego, La Jolla, California, USA; 2 Department of Psychiatry and Psychotherapy, Division of Biological Psychiatry, Medical University of Vienna, Vienna, Austria; 3 UCSD Eating Disorder Research and Treatment Program, UCSD Department of Psychiatry, 4510 Executive Dr., Suite 315, San Diego, CA 92121-3021 USA

**Keywords:** Anticonvulsants, Binge eating, Purging, Emotion dysregulation, Lamotrigine

## Abstract

**Background:**

There is little effective psychopharmacological treatment for individuals with eating disorders who struggle with pervasive, severe affective and behavioral dysregulation.

**Methods:**

This pilot open series evaluated lamotrigine, a mood stabilizer, in the treatment of patients with eating disorders who did not respond adequately to antidepressant medications. Nine women with anorexia nervosa- or bulimia nervosa-spectrum eating disorders in partial hospital or intensive outpatient dialectical behavior therapy (DBT)-based eating disorder treatment took lamotrigine for 147 ± 79 days (mean final dose = 161.1 ± 48.6 mg/day). Participants completed standardized self-report measures of emotion dysregulation and impulsivity after lamotrigine initiation and approximately biweekly thereafter. Mood and eating disorder symptomatology were measured at lamotrigine initiation and at time of final assessment.

**Results:**

Lamotrigine and concurrent DBT were associated with large reductions in self-reported affective and behavioral dysregulation (*p*s < 0.01). Eating disorder and mood symptoms decreased moderately.

**Conclusions:**

Although our findings are limited by the confounds inherent in an open series, lamotrigine showed initial promise in reducing emotional instability and behavioral impulsivity in severely dysregulated eating-disordered patients. These preliminary results support further investigation of lamotrigine for eating disorders in rigorous controlled trials.

## Background

Bulimia nervosa (BN) and the binge-eating/purging subtype of anorexia nervosa (AN-BP) are characterized by loss-of-control eating and compensatory behaviors (e.g., self-induced vomiting) and are associated with significant medical morbidity and chronicity [1, 2]. Other impulsive behaviors, including non-suicidal self-injury, shoplifting, and drug and alcohol abuse, often are comorbid with these eating disorders [3–5]. Selective serotonin reuptake inhibitors (SSRIs) have been the mainstay of psychopharmacological treatment of BN since FDA approval of fluoxetine more than 20 years ago [6]. Although no medications are approved for AN-BP, SSRIs frequently are tried in these patients as well [7]. Still, first-line interventions, including both SSRIs and behavioral therapies such as cognitive-behavioral therapy (CBT), are ineffective for a large proportion of patients with BN and AN-BP who have been described as “multi-impulsive,” and struggle with a range of dysregulated behaviors [8–12].

A growing body of evidence suggests that these dysregulated behaviors may be linked to emotional instability, and that pervasive deficits in cognitive and behavioural self-regulatory control may contribute to eating disorder behaviors and inadequate response to existing eating disorder treatments [12–19]. Self-reported emotion regulation problems have been associated with eating disorder cognitions and compensatory behaviors in BN [13]. Further, increasing negative affect and decreasing positive affect often precede binge eating and purging [20–23], and affective instability is associated with more frequent weight loss behaviors in AN [24] and more frequent bulimic behaviors in BN [25]. Extreme increases in negative affect are less likely after bulimic behaviors, but average affective instability is worse after bulimic episodes in BN [26]. As such, the powerful but only temporary relief of dysregulated and impulsive behaviors may ultimately reinforce maladaptive cycles [27], and affect dysregulation may contribute to AN-BP, BN, and bulimic symptoms more broadly. These data suggest that directly targeting regulatory deficits may be key to more effective treatment.

Mood-stabilizing medications have been shown to reduce affective and behavioral dysregulation in other psychiatric populations. One such medication is lamotrigine, an antiepileptic drug. It has received FDA indication [28] for maintenance treatment of bipolar I disorder to delay the time to occurrence of mood episodes, and it is used widely for bipolar II disorder and unspecified bipolar and related disorders [29]. Lamotrigine also has shown promise in the treatment of borderline personality disorder (BPD) [30, 31]. Data from two small randomized-controlled trials in BPD indicated that lamotrigine was superior to placebo in reducing anger [32], affective instability, and impulsivity (including symptoms of BN) [33]. A much larger multi-center RCT of lamotrigine for long-term treatment of BPD is in progress [34].

Consistent with the literature, our clinical experience is that severely dysregulated eating-disordered patients often show little or no response to antidepressant monotherapy, and in some cases, they appear to become more agitated with this treatment. This led us to speculate that medications with mood-stabilizing properties [32–34] may be a better alternative for some. We previously reported a positive response to lamotrigine in five patients with eating disorders characterized by bulimic symptoms and significant affective and behavioral dysregulation [35]. Though encouraging, these case reports were based on personal observation. To support a potential future controlled trial of lamotrigine, the current study aimed to confirm our observations in a larger series of patients, utilizing standardized instruments [36, 37] designed to assess changes in affective and behavioral dysregulation in response to treatment, as well as mood and eating disorder symptomatology.

## Methods

### Participants

Participants enrolled in this open trial were female patients in the UCSD Eating Disorders Program deemed appropriate for lamotrigine initiation (*n* = 14) based on presence of pervasive emotion dysregulation, poor impulse control, and endorsed binge eating and/or purging, as clinically assessed by the program psychiatrists. All enrolled patients had a prior history of inadequate response to treatment with antidepressants. Upon entering the study, patients were continued on other current medications where considered appropriate by the psychiatrist (for example, if there had been a partial response, or for treatment of co-morbid conditions). Participants included in this report took lamotrigine for a minimum of 60 days. This is because lamotrigine requires a very gradual titration for safety reasons (see Discussion), so significant results may take longer to realize than in most medication trials.

### Procedure

#### Lamotrigine titration

Participants started at a dose of 25 mg/day for two weeks, then increased to 50 mg/day for the next two weeks. Subsequent rate of titration was variable, with a maximum increase of 50 mg/day every two weeks until reaching a therapeutic dose (expected range from 100 mg/day to 300 mg/day). Increases and maximum dose were determined by the psychiatrist based on tolerability and therapeutic response.

#### Additional treatment as usual

All participants entered treatment at the partial hospital program (PHP) level (10 h per day, six days per week). With clinical improvement, patients stepped down to 6 h per day at the PHP level, and ultimately to 4 h per day, three days per week in the intensive outpatient program (IOP). All patients received Dialectical Behavior Therapy (DBT) [38], adapted for the PHP/IOP setting, throughout their course of treatment at UCSD. This included weekly individual DBT sessions, twice weekly skills training groups using the DBT Skills manual [39], other groups based on DBT principles (e.g., behavioral chain analysis), and skills coaching via phone or text messaging outside of program hours. All therapists participated in a weekly DBT consultation team [38].

The Human Research Protections Program at the University of California, San Diego approved of the collection of data for this study. All participants provided written informed consent before completing assessments and consented to treatment including psychotropic medication.

### Measures

Participants completed assessments of emotional and behavioral dysregulation at baseline and approximately every two weeks thereafter (mean time between assessments = 20.5 days, *SD* = 12.9 days) for up to seven additional time points after lamotrigine initiation. Our primary outcome measures were well-validated assessments of weekly changes in cognitive, affective, and behavioral dysregulation, which originally were designed to track symptoms of borderline personality disorder (BPD):

#### Borderline evaluation of severity over time (BEST) [36]

The BEST was developed to rate the thoughts, emotions, and behaviors typical of BPD The scale includes 15 items and three subscales. Eight items assess cognitive and affective dysregulation, four items assess behavioral dysregulation, and three items assess skillful behavioral regulation. All items are rated on a 5-point Likert-type scale ranging from 1 to 5. The BEST has been shown to have good to excellent internal consistency, both in individuals with BPD and a comparison sample, and moderate test-retest reliability [36].

#### Zanarini rating scale for borderline personality disorder (ZAN-BPD) [40]

The ZAN-BPD is a clinician-administered scale for the assessment of change in borderline psychopathology over a 2-week time period. Each of the nine criteria for BPD is rated on a 5-point anchored rating scale of 0 to 4, yielding a total score of 0 to 36. The ZAN-BPD includes three items assessing affective dysregulation, two items assessing cognitive dysregulation, two items assessing impulsive [41] behaviors, and two items assessing unstable interpersonal relationships. For feasibility reasons, the ZAN-BPD was administered as a self-report questionnaire; however, the clinician-administered version of the ZAN-BPD has shown good internal consistency, with test-retest reliability in the good to excellent range [40].

Secondary outcome measures, administered at treatment initiation and at the time of final assessment, included ratings of eating disorder symptom severity, anxiety, and depression:

#### Eating disorder examination questionnaire (EDE-Q) [41]

The EDE-Q is a 36-item self-report questionnaire adapted from the investigator-based Eating Disorder Examination (EDE) [42]. The EDE-Q consists of four subscales, including Restraint, Shape Concern, Weight Concern, and Eating Concern, and assesses eating disorder symptomatology during the past 28 days. Items of all four subscales were rated on a 7-point Likert-type scale ranging from 0 to 6. The EDE-Q subscales have demonstrated acceptable internal consistency and good test-retest reliability and convergent and discriminant validity [43, 44].

#### State-trait anxiety inventory (STAI) [45]

The STAI is a 40-item self-report measure, including 20 items assessing trait anxiety and 20 assessing state anxiety. All items are rated on a 4-point Likert-type scale ranging from 1 to 4, with higher scores indicating greater anxiety. The STAI has been shown to have excellent internal consistency in large samples [46].

#### Beck depression inventory (BDI-II) [47]

The BDI-II is a self-report questionnaire that measures severity and symptoms of depression. The questionnaire includes 21 items, and items are rated on a 4-point Likert-type scale ranging from 0 to 3, with higher scores indicating greater severity of depression. The BDI-II has demonstrated excellent internal consistency and high convergent validity [48], as well as excellent test-retest reliability [49].

### Statistical analysis

Related-samples Wilcoxon signed rank tests were used to examine changes from baseline to end of treatment in BEST and ZAN-BPD scores. Average BEST and ZAN-BPD scores did not differ across diagnoses; therefore, diagnosis was not included in the final models. Cohen’s *d* [50] was calculated to measure effect size and estimate the magnitude of lamotrigine effectiveness in this sample. Additionally, a reliable change index (RCI) [51] was used to determine whether the symptom reductions measured by the BEST and the ZAN-BPD were clinically significant and statistically reliable (RCI cut-off: ≥ 1.96). RCI was calculated for each patient (the difference between baseline and final assessment score divided by the standard error of difference between the two scores) on the BEST and the ZAN-BPD. In exploratory analyses, related-samples Wilcoxon signed rank tests were used to examine changes from baseline to end of treatment in eating disorder, depression, and anxiety scores.

## Results

### Participants

**Fig. 1:**
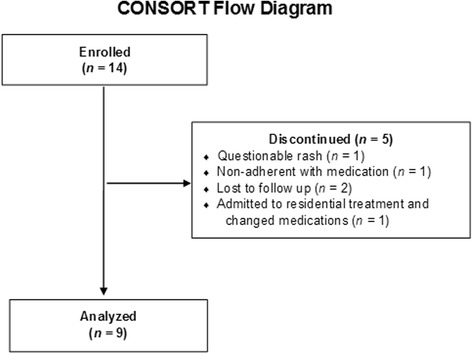
CONSORT Flow Diagram for Lamotrigine Open Trial. Out of 14 enrolled patients, five were discontinued from the trial: one patient stopped lamotrigine after she developed a possible rash, one non-adherent participant reported to psychiatrists several months into the trial that she never started taking lamotrigine, and three were lost to follow up after discharging prematurely from our program. As none of the five discontinued patients completed 60 days of lamotrigine titration, they were excluded from the analysis. The final sample included nine patients who started lamotrigine at UCSD and took the medication for at least 60 days

Of the 14 participants initially enrolled in this open trial, five were excluded from analyses because they did not complete 60 days of lamotrigine treatment and self-report assessments (Fig. 1). The nine participants who were treated for 60 or more days (Table 1) were women ranging in age from 18 to 42 years (*M* = 30.1 years, *SD* = 7.8), with a mean intake BMI of 22.6 kg/m^2^ (*SD* = 3.3). Average length of time on lamotrigine was 147.4 days (*SD* = 78.9). Mean dose at time of final assessment was 161.1 mg/day (*SD* = 48.6), with a range of 100 mg/day to 200 mg/day. Baseline characteristics and medication information for each individual are presented in Table 1. In most instances, titrations of concurrent medications were complete before initiation of lamotrigine. In three cases, other medications were changed over the course of lamotrigine treatment. One patient changed antidepressants (duloxetine was replaced with sertraline and subsequently discontinued; see Table 1 footnote), and two patients discontinued other medications (one patient discontinued naltrexone, bupropion XL, and trazodone during her first 2–3 months in the program; one patient discontinued trazodone during her first 1–2 months in the program).

**Table 1:** Sample Characteristics

ID	Age	Ethnicity	Race	BMI (kg/m2)	Diagnosis at Admission	Days on Lamotrigine at Baseline Assessment	Total Days on Lamotrigine	Final Lamotrigine Dose (mg/day)	Concurrent Medications
1	28	Non-Hispanic	Black	26.3	EDNOS^a^	−2	223	150	quetiapine XR, bupropion XL, levomilnacipran
2	23	Non-Hispanic	White	20.2	EDNOS^b^	−5	246	200	duloxetine, trazodone
3	41	Non-Hispanic	White	21.9	EDNOS^c^	−4	253	100	escitalopram
4	42	Hispanic	Other	28.3	BN	1	190	100	venlafaxine XR, naltrexone
5	18	Non-Hispanic	White	19.7	AN-BP	1	86	200	sertraline, gabapentin
6	31	Non-Hispanic	White	20.1	AN-BP	1	85	200	gabapentin, naltrexone
7	25	Non-Hispanic	White	19.1	AN-R	0	102	200	duloxetine, sertraline^d^
8	31	Hispanic	Other	22.3	AN-BP	0	71	100	fluoxetine
9	32	Non-Hispanic	Other	25.7	BN	1	71	200	duloxetine

^a^High-normal to mildly overweight with restrictive eating and purging; ^b^Mildly underweight AN with alternating severe restrictive eating, bingeing, and occasional purging; ^c^Lifetime history of mostly BN with episodes of AN-BP. During period of study, most characteristic of low-normal-weight AN-P; ^d^Duloxetine was concurrent with lamotrigine for one month and was subsequently replaced with sertraline in the second month. In the third month, lamotrigine was the only medication prescribed. BMI = body mass index; AN-BP = anorexia nervosa, binge-eating/purging subtype; EDNOS = eating disorder not otherwise specified; BN = bulimia nervosa

Average length of stay in the eating disorders program (including both PHP and subsequent IOP) was 186 calendar days (*SD* = 39.72). During admission, the patients spent an average of 82.30 days (*SD* = 34.12) attending treatment groups at our facility (as noted previously, patients attended for 3–6 days during each calendar week, depending upon level of care). Four of the nine patients included in the analyses completed follow-up assessments after discharge. These patients were discharged from the treatment program on lamotrigine day 124, 162, 69, and 56. Three of these four participants completed 3-month follow-up assessments, and one completed a 6-month follow-up assessment.

### Changes in affective and behavioral dysregulation

**Fig. 2:**
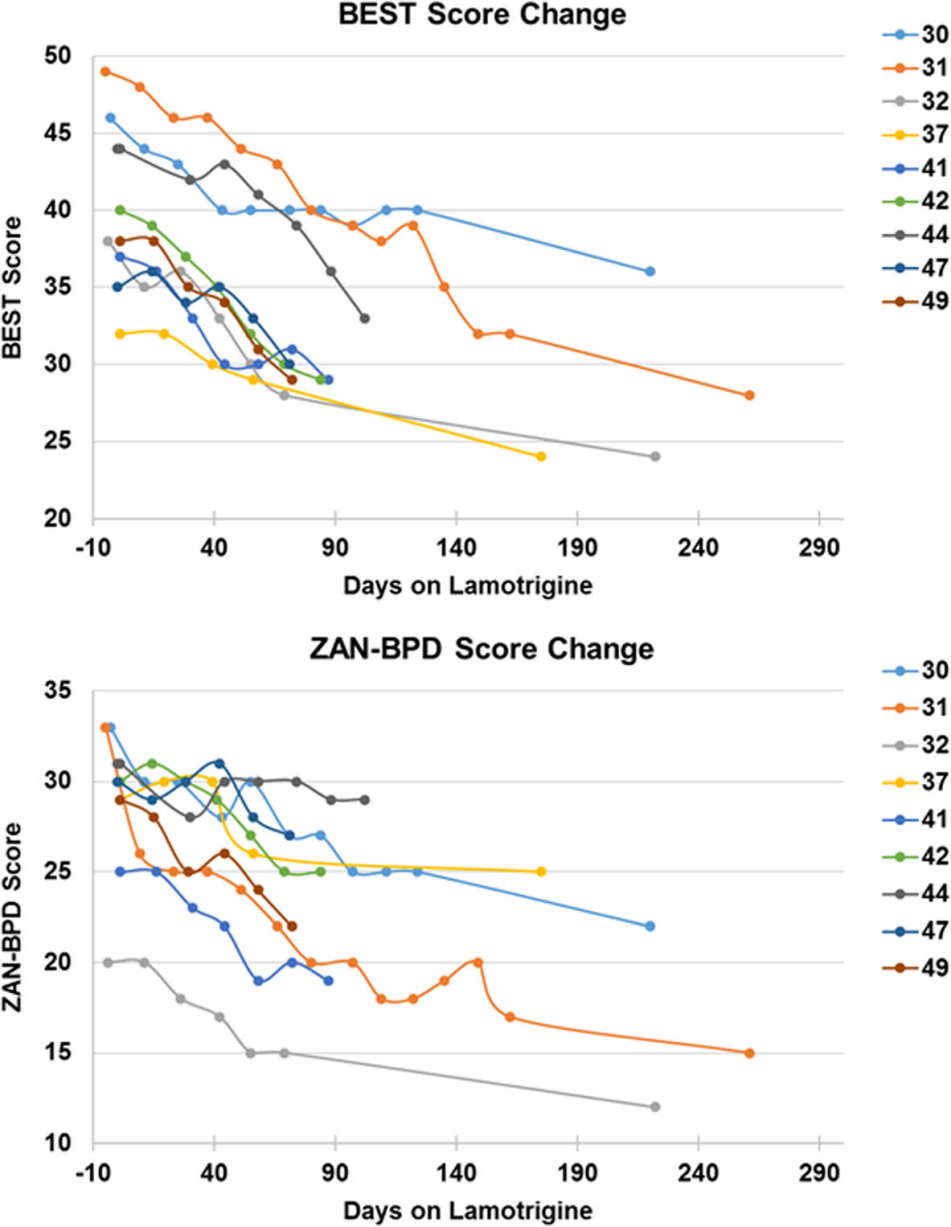
BEST and ZAN-BPD Score Change Over Time. The Borderline Evaluation of Severity Over Time (BEST) score change over time is presented in panel **a** and the Zanarini Rating Scale for Borderline Personality Disorder (ZAN-BPD) score change is shown in panel **b**. Days on lamotrigine at each assessment point are shown on the x axis and scores are shown on the y axis

BEST and ZAN-BPD scores over time are plotted in Fig. 2. Pre- to post-treatment reductions in dysregulation as measured by the BEST (z = 2.670, *p* = 0.008) and ZAN-BPD (z = 2.666, *p* = 0.008) were statistically significant (Table 2). At one month of lamotrigine treatment, BEST score reduction was very large (*d* = 2.41) and ZAN-BPD score reduction was moderate to large (*d* = 0.78). As depicted by the graphs in Fig. 2, patients appeared to continue improving several months into lamotrigine treatment with further dose titration. Effect sizes for baseline to end-of-assessment reductions on the ZAN-BPD (Cohen’s *d* = 1.53) and on the BEST (Cohen’s *d* = 2.29) were very large.

**Table 2:** Scores on Secondary Outcome Measures Before and After Lamotrigine Treatment

Measure	Pre *M (SD)*	Post *M (SD)*	*p*	Cohen’s *d*
BEST	39.89 (5.46)	29.11 (3.82)	0.008	2.29
ZAN-BPD	28.89 (4.11)	21.11 (5.90)	0.008	1.53
EDE-Q Scores				
Restraint	2.8 (1.5)	1.3 (0.9)	0.058	1.21
Eating Concern	2.9 (1.7)	2.2 (1.4)	0.093	0.45
Shape Concern	4.7 (1.2)	3.3 (1.6)	0.093	0.99
Weight Concern	4 (1.8)	3.4 (1.5)	0.406	0.36
Global	3.7 (1.4)	2.7 (1)	0.051	0.82
BDI-II	30.7 (15.4)	24.2 (12.4)	0.314	0.46
STAI State	60.2 (11.4)	60.2 (12.1)	0.799	0
STAI Trait	54 (8.9)	56.1 (10.6)	0.373	−0.21

Note: Presented *p* values represent the results of exploratory related-samples Wilcoxon signed rank tests; EDE-Q = Eating Disorder Examination Questionnaire; STAI = State-Trait Anxiety Inventory; BDI = Beck Depression Inventory

Most of the patients (77.8%) showed a clinically significant and reliable treatment response (RCI > 1.96) as measured by the ZAN-BPD (mean RCI = 4.46, *SD* = 3.34), and roughly half (55.6%) of the patients showed a clinically significant and reliable treatment response as measured by BEST scores (mean RCI = 2.26, *SD* = 0.96).

### Changes in mood, anxiety, and eating disorder symptoms

Results of exploratory analyses examining changes in other symptoms are presented in Table 2. Despite medium to large reductions in EDE-Q Restraint (*d* = 1.21), Eating Concern (*d* = 0.45), Shape Concern (*d* = 0.99), and EDE-Q Global (*d* = 0.82) scores, these pre- to post-treatment differences did not reach statistical significance. Reductions in depression scores were small to medium (0.46), but changes in anxiety scores were negligible.

## Discussion

This is the first study to use standardized measures of affective and behavioral dysregulation to document lamotrigine response in eating-disordered patients over a substantial time period. Lamotrigine and concurrent DBT were associated with significant and medium to large self-reported reductions in dysregulated emotions and problems with impulse control. Further, in our small sample, we found preliminary evidence of reduced eating disorder symptoms and depression, but little change in anxiety symptoms. Because patients received multimodal eating disorder treatment during lamotrigine titration, these data cannot isolate the effects of the medication. However, our preliminary findings suggest that the targeted effects of lamotrigine in eating disorder populations warrant further investigation.

Our results are consistent with prior reports of lamotrigine treatment benefit for some patients with BN- and AN-BP-spectrum disorders [35, 52, 53] and for some patients with binge-eating behaviors [54]. As noted previously, lamotrigine is utilized in other conditions characterized by dysregulated mood and poor impulse control, including bipolar disorder and BPD. When used in bipolar disorder, it is most notably effective in reducing depressive symptoms [29]. This ability to stabilize mood with potentially greater impact on depression [55–57] may account for our exploratory and very preliminary findings of moderate reductions in depressive symptoms for some eating-disordered patients.

The literature on affective instability suggests some blurring of categorical lines between these diagnoses. While it is known that bipolar disorder and BPD can co-exist, and that patients with bipolar disorder alone may display affective dysregulation not unlike that in BPD [58, 59], more recent real-time data collection suggests a similar type of affective instability in patients with BN, supporting the possibility of a transdiagnostic rather than disorder-specific mechanism [60]. Although determining a specific DSM diagnosis for the dysregulated characteristics of eating-disordered patients can be difficult, previous reports have suggested that up to 68% of those with eating disorders may have bipolar disorder if the so-called “soft spectrum” is included [61–63]. Additionally, 14% to 35% of patients with BN are believed to have BPD [64–67]. Finally, up to 50% of individuals across the range of eating disorders are estimated to abuse alcohol or other illicit substances [4, 5]. Not uncommonly, patients with AN-BP and BN-spectrum disorders struggle with a combination of these problems. Clinical evaluation by our psychiatrists (MET, UFB) suggested that although participants reported significant difficulties with mood regulation and impulse control, most did not meet full criteria for BPD diagnosis. Self-report measures assessed changes in affect regulation and impulse control in response to treatment, but diagnostic category was not included in study entry criteria nor was it a measure of treatment response. Administration of structured research interviews to assess for personality disorders was not feasible in our clinical setting. Future studies should include structured diagnostic interviews to determine the impact of lamotrigine on dysregulation in eating-disordered individuals with and without comorbid BPD.

Lamotrigine appears to be acceptable to many patients because of its typically low side effect burden [68] and reported weight neutrality (the latter of which may be extremely important for medication adherence in those with eating disorders). Although lamotrigine usually is well-tolerated, as was the case in our trial, there are precautions to be followed and occasional drawbacks with its use. The most common potential side effects include benign rash (up to 10%), headache, nausea, insomnia, somnolence, fatigue, dizziness, blurred vision, ataxia, tremor, rhinitis, and abdominal pain. One of our 14 participants discontinued because of a possible benign rash, which is the most frequent cause of discontinuation of lamotrigine in general [69]. This is because if any skin eruption is suspected of being a drug-induced rash, the medication should be stopped, with the usual recommendation that it not be retried in the future. Such precautions are taken because a rare but very serious adverse effect of lamotrigine is the rash of Stevens-Johnson syndrome and epidermal necrolysis [70]. In studies with epilepsy patients, incidence of Stevens-Johnson syndrome varied between 0.08% and 0.3% in adults [69]. A slow titration is necessary to minimize the risk of rash, which may substantially delay optimal effects for some patients. As an additional dosing consideration, the patients in our trial were not taking oral contraceptives, but it is important to keep in mind that these medications can decrease concentrations of lamotrigine [71].

Lamotrigine is a glutamate antagonist, believed to stabilize mood by inhibiting release of this excitatory neurotransmitter [57]. Our findings raise the question as to whether glutamatergic abnormalities play a role in affective and behavioral dysregulation in individuals with eating disorders as they may in bipolar disorder and BPD [72, 73]. This could help explain why traditionally-used serotonergic antidepressant monotherapy has limited impact for many patients with AN-BP- and BN-spectrum disorders.

Lamotrigine may specifically target corticolimbic circuit alterations that contribute to affective dysregulation. Several functional magnetic resonance imaging (fMRI) studies have suggested that relative to healthy controls, individuals with bipolar disorder, BPD and BN all show increased amygdala activation and decreased activation in dorsolateral prefrontal cortex (DLPFC) in response to negative emotional stimuli [74–76]. Successful modulation of emotional responses is partially dependent on adequate DLPFC and ventromedial prefrontal cortex (VMPFC) signalling [77]. In healthy individuals, lamotrigine, in combination with prefrontal transcranial magnetic stimulation, increases prefrontal circuit connectivity [78]. Functional imaging studies in bipolar disorder similarly suggest lamotrigine response is associated with increased PFC activation and decreased amygdala activation to negatively-valenced emotional stimuli [79, 80]. These corticolimbic changes are believed to be mediated by a reduction in glutamate [57, 81]. Research integrating fMRI with positron emission tomography is needed to test this hypothesis in eating disorders.

### Strengths and limitations

Our study is subject to several limitations. First, almost all of the patients concurrently were taking other medication, most notably antidepressants. Therefore, it is not possible to isolate the effects of lamotrigine relative to other medications. Second, and perhaps the greatest confounding factor, was the comprehensive concurrent DBT treatment and/or the structure provided by a PHP/IOP. The design of this pilot study cannot parse the relative impact of these other aspects of treatment from those of lamotrigine. Third, all of our patients were female, and future study in male patients is needed. Fourth, we did not assess plasma levels of lamotrigine. Finally, effect size for changes in depression and anxiety also may have been impacted by the second and final assessment time point for these measures occurring near the date of program discharge for the majority of patients. These final assessment scores may reflect 1) temporarily worsened depression and anxiety, as the uncertainties and insecurities associated with treatment termination may be particularly pronounced in patients who struggle with emotion regulation [82], or 2) increased emotional awareness with DBT, which can elevate depression and anxiety scores despite reduction in behavioral symptoms [83, 84].

Despite these limitations, our pilot open series has important strengths. Many of the participants had a history of multiple unsuccessful medication trials, all had been poorly responsive to antidepressant monotherapy before entering the trial, and, anecdotally, they often described an improved ability to utilize DBT and other emotion regulation strategies during and following titration of lamotrigine specifically. Furthermore, 7 of the 9 patients (77.8%) had prior exposure to DBT and all had prior exposure to structured treatment either in our program or at other locations prior to lamotrigine initiation. Although this included high levels of care for most (residential or inpatient treatment for 8 out of 9 patients), none had shown significant improvement from those factors alone.

The treatment effect as measured by the ZAN-BPD was comparable to that observed in a prior trial of lamotrigine (*d* = 1.40; [33]) and the treatment effect as measured by the BEST was superior to that reported in a trial of Systems Training for Emotional Predictability and Problem Solving (*d* = 0.50; [36]). In addition, aspects of our data very preliminarily suggest a potential added benefit of lamotrigine: Four patients included in our sample continued to show symptom improvement after discharge (i.e., after completing concurrent intensive DBT).

## Conclusion and future directions

In summary, our initial data support further study of lamotrigine for the treatment of dysregulation in eating-disordered patients. Results from our small sample must be interpreted with caution. It is premature to propose that lamotrigine is a treatment for dysregulated mood and impulse control in eating disorders. Nevertheless, our findings preliminarily suggest that directly targeting regulatory deficits may be key to more effective treatment and support the feasibility of studying lamotrigine efficacy in eating-disordered populations. Our pilot findings are perhaps most important in supporting the need for large-scale, rigorously controlled investigations of lamotrigine, used with or without concurrent DBT or other therapies, to elucidate how these factors might interact to treat dysregulated behavior in eating disorders.

## Abbreviations

AN: Anorexia nervosa
AN-BP: Anorexia nervosa, binge-eating/purging type
BDI-II: Beck Depression Inventory
BEST: Borderline Evaluation of Severity Over Time
BMI: Body mass index
BN: Bulimia nervosa
BPD: Borderline personality disorder
DBT: Dialectical behavior therapy
DLPFC: Dorsolateral prefrontal cortex
EDE: Eating Disorder Examination
EDE-Q: Eating Disorder Examination Questionnaire
EDNOS: Eating disorder not otherwise specified
fMRI: Functional magnetic resonance imaging
IOP: Intensive outpatient program
PHP: Partial hospital program
RCI: Reliable change index
SSRI: Selective serotonin reuptake inhibitors
STAI: State-Trait Anxiety Inventory
VMPFC: Ventromedial prefrontal cortex
ZAN-BPD: Zanarini Rating Scale for Borderline Personality Disorder
